# Beneficial Effects of Dietary Flaxseed on Non-Alcoholic Fatty Liver Disease

**DOI:** 10.3390/nu16040466

**Published:** 2024-02-06

**Authors:** Mihir Parikh, Broderick C. Hirst, Kimberley A. O’Hara, Thane G. Maddaford, J. Alejandro Austria, Aleksandra Stamenkovic, Liping Yu, Branislav Kura, Bhavana Garg, Thomas Netticadan, Spencer D. Proctor, Grant N. Pierce

**Affiliations:** 1 Department of Physiology and Pathophysiology, Faculty of Health Sciences, University of Manitoba, Winnipeg, MB R3E 0W3, Canadatnetticadan@sbrc.ca (T.N.); 2 Canadian Centre for Agri-Food Research in Health and Medicine (CCARM), Winnipeg, MB R2H 2A6, Canada; 3 The Institute of Cardiovascular Sciences, Winnipeg, MB R2H 2A6, Canada; 4 Agriculture and Agri-Food Canada, St. Boniface Hospital Albrechtsen Research Centre, 351 Taché Avenue, Winnipeg, MB R2H 2A6, Canada; 5 Institute for Heart Research, Centre of Experimental Medicine, Slovak Academy of Sciences, 841 04 Bratislava, Slovakia; 6 Metabolic and Cardiovascular Diseases Laboratory, Division of Human Nutrition, University of Alberta, Edmonton, AB T6G2P5, Canada; spencer.proctor@ualberta.ca

**Keywords:** flaxseed, JCR:LA-*cp* rats, diet, fatty liver disease, NAFLD, obesity, cholesterol

## Abstract

Non-alcoholic fatty liver disease (NAFLD), a significant cause of chronic liver disease, presents a considerable public health concern. Despite this, there is currently no treatment available. This study aimed to investigate dietary flaxseed in the JCR:LA-corpulent rat strain model of NAFLD. Both obese male and female rats were studied along with their lean counterparts after 12 weeks of ingestion of a control diet, or control diet with flaxseed, or high fat, high sucrose (HFHS), or HFHS plus flaxseed. Obese rats showed higher liver weight and increased levels of cholesterol, triglyceride, and saturated fatty acid, which were further elevated in rats on the HFHS diet. The HFHS diet induced a significant two-fold elevation in the plasma levels of both aspartate aminotransferase and alanine aminotransferase in the obese male and female rats. Including flaxseed in the HFHS diet significantly lowered liver weight, depressed the plasma levels of both enzymes in the obese male rats, and reduced hepatic cholesterol and triglyceride content as well as improving the fatty acid profile. In summary, including flaxseed in the diet of male and female obese rats led to an improved lipid composition in the liver and significantly reduced biomarkers of tissue injury despite consuming a HFHS chow.

## 1. Introduction

The most prevalent chronic liver disease globally is non-alcoholic fatty liver disease (NAFLD) [1,2,3], with a world incidence of approximately 25% [2,3,4,5]. NAFLD incidence appears to be more frequently found in males as compared to females [2,3,6,7], with incidence levels as much as six times higher in adolescent males compared to adolescent females [8]. With increasing levels of obesity and diabetes, two important risk factors for NAFLD, expected to increase in the years to come, the incidence of NAFLD will be even higher [2,3,4,9]. Obesity, insulin resistance, and metabolic syndrome (central obesity, hyperglycemia, hypertension, and dyslipidemia) are the most commonly recognized risk factors for NAFLD. 

Unfortunately, to date, there is no known pharmacological therapy for NAFLD [10]. The use of an animal model to test strategies to prevent or inhibit the development of NAFLD would be a useful step forward in research on NAFLD. The JCR:LA corpulent rat exhibits almost all of the same metabolic indicators as NAFLD and could be an ideal animal model for NAFLD. These animals display severe obesity, dyslipidemia, hepatic disease, hyperglycemia when challenged with a glucose load, and insulin resistance [11,12]. Common co-morbidities for NAFLD like heart disease and kidney disease are also present in the JCR:LA-*cp* rat [13,14]. Therefore, the JCR:LA-*cp* rat may be an ideal model in which to study the efficacy of any novel interventions to treat NAFLD. In view of the clear association of NAFLD with diet, it is reasonable to propose that a dietary intervention may be a particularly effective therapeutic approach. 

The inclusion of flaxseed in the diet has beneficial effects on a variety of organs including the vascular system and the heart [15]. Flaxseed is a rich source of the omega-3 fatty acid alpha-linolenic acid (ALA), antioxidant lignans, and fibre [15,16,17]. Because NAFLD has aspects of liver inflammation, oxidation, and fibrosis involved in its pathogenesis [18,19], these pathological characteristics may be addressed by the components in flaxseed. ALA has anti-inflammatory actions [15], lignans are antioxidants [15], its fibre lowers plasma lipids levels [17], and flaxseed can also reduce tissue fibrosis [20]. This raises the distinct possibility that flaxseed may be of benefit in a model of NAFLD. The purpose of the present study is to evaluate the effects of dietary flaxseed supplementation, a high-fat, high-sucrose diet supplementation, or a flaxseed plus high-fat, high-sucrose enriched diet on liver pathology in JCR:LA corpulent rats and their corresponding lean animals of both sexes. 

## 2. Experimental Design, Materials and Methods

### 2.1. Experimental Design

**Figure 1:**
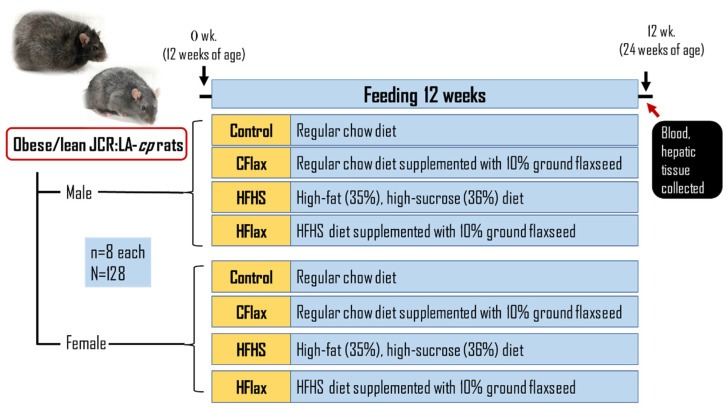
Illustration of the experimental design. Con: control diet; CFlax: flaxseed-supplemented diet; HFHS: high-fat, high-sucrose-supplemented diet; HFlax: high-fat, high-sucrose and flaxseed-supplemented diet.

Male and female obese JCR:LA-*cp* rats and their genetic control lean JCR:LA-*cp* rats were randomized into groups (*n* = 8 each) to receive, for 12 weeks, either (a) control diet (Con), (b) control diet supplemented with 10% ground flaxseed (CFlax), (c) a high-fat/high-sucrose (HFHS) diet, or (d) HFHS supplemented with 10% ground flaxseed (HFlax). Prolab^®^ RMH 3000 (TestDiet, Richmond, IN, USA) regular rodent chow was the control diet. The HFHS diet from TestDiet, Richmond, IN, USA contained AIN-93G chow with 35% fat (lard) and 36% carbohydrate (mostly sucrose). BakePur milled flaxseed was gratefully received from Pizzey Ingredients, Russell, Manitoba, Canada. The rats were allowed ad libitum access to water and food. Food intake was measured daily and averaged to obtain weekly values. Body weights were weighed weekly. The dietary protocol was completed at 12 weeks when the JCR:LA-cp rats were 24 weeks old, Figure 1. The nutritional composition of all of the experimental diets (ash, protein, fibre, carbohydrate, protein, fat, energy) has been reported previously in detail [13]. Body weights for all groups throughout the course of the study have also been reported previously [13]. 

### 2.2. Biological Sample Collection and Analysis

After anesthesia, the fasted blood sample was collected from the inferior vena cava by opening the thoracic cavity and the heart was immediately excised. Livers were isolated, rinsed in PBS, weighed, and then stored at −80 °C for subsequent analysis. The plasma was separated by centrifugation (3000 RPM, 4 °C for 15 min) and stored at −80 °C for subsequent biochemical analysis. The fasted plasma biochemical profile was assessed using cobas c 111 (Roche Diagnostics, Indianapolis, IN, USA) for triglycerides (TG), alanine aminotransferase (ALT), and aspartate aminotransferase (AST). Plasma cholesterol levels were measured spectrophotometrically using the ThermoFisher (Middletown, VA, USA) total cholesterol kit #TR13421, as previously described [13].

Total fatty acids were extracted from the liver tissue and plasma samples and derivatized as previously described [13,17]. The fatty acid methyl esters were separated on a DB225MS column (30 m × 0.25 mm diameter and 0.25 μm film thickness; Agilent Technologies Canada Inc., Mississauga, ON, Canada) using a Varian 450 GC with FID. The temperature program was 70 °C for 2 min, the temperature was raised to 180 °C at 30 °C/minute, held for 1 min, raised to 200 °C at 10 °C/min, held for 2 min, then raised to 220 °C at 2 °C/min and held for 10 min before finally raising to 240 °C at 20 °C/min and holding for 15 min. Total run time was 46.67 min, and samples were run with a 20:1 split ratio and a 1.3 mL/min column flow. Hydrogen was used as carrier gas. Components were identified by comparison with authentic standards (Nu-Chek Prep, Elysian, MN, USA).

The quantification of liver triglyceride content was carried out after homogenization of 50 mg of tissue in 300 μL of 1X NP40 Substitute Assay Reagent using an Omni Bead Ruptor 24 (Omni Inc., Kennesaw, GA, USA) with Hard Tissue Homogenizing Mix 2.8 mm Ceramic (2mL Reinforced Tube). After centrifugation at 10,000× *g* for 10 min at 4 °C, the supernatant, containing both soluble and insoluble components, was collected and frozen at −80 °C for future analysis. Liver samples were analyzed using Cayman Chemical’s Triglyceride Colorimetric Assay Kit (Cayman Chemical, Ann Arbor, MI, USA).

Liver total cholesterol content was assessed after homogenizing 30 mg of tissue with 300 μL of chloroform:isopropanol:NP-40 (7:11:0.1) using an Omni Bead Ruptor 24 (Omni International Inc., Kennesaw, GA, USA). The resulting extract was centrifuged at 15,000× *g* for 10 min at 4 °C and the organic phase was collected, evaporated using nitrogen, and then dissolved in 600 μL of 1X Assay Diluent using sonicating and vortexing for homogeneity. The dissolved extract was assayed using Cell Biolabs Inc., (San Diego, CA, USA) Total Cholesterol Assay Kit (Colorimetric) (#STA-384), following the standard protocol.

### 2.3. Hepatic Protein Expression

Protein expression of liver sterol regulatory element-binding transcription factor 1 (SREBP-1), 3-hydroxy-3-methylglutaryl coenzyme-A reductase (HMGCR), cytochrome P450 7A1 (CYP7A1), and CD36 (also known as cluster of differentiation 36 or fatty acid translocase (FAT)) were measured by western blotting as described in detail elsewhere [9]. The primary antibodies used were obtained from SREBP-1 (A-4) (Santa Cruz, CA, USA), HMGCR, CYP7A1, and CD36 (Abcam, Waltham, MA, USA). A BLUeye Prestained Protein Ladder was used to approximate kDa. BioRad 4–15% gradient precast gels were used for each western blot. Blots were imaged using the ChemiDoc Technology (BioRad, Hercules, CA, USA) after applying substrate. 

### 2.4. Statistical Analysis

Statistical analysis was as described in detail [13]. Briefly, data were analyzed by two-factor ANOVA with sex and diet as the independent variables. Post hoc analysis was carried out via the Student–Newman–Keuls test and Tukey’s test. Statistical significance was determined at a *p* level < 0.05. Because of the complexity of the analysis and the comparisons, we have restricted our focus to identifying significant differences: (i) versus respective control group, (ii) versus respective HFHS group, and (iii) obese vs. lean within same respective dietary group. 

## 3. Results

### 3.1. Liver Weight

**Figure 2:**
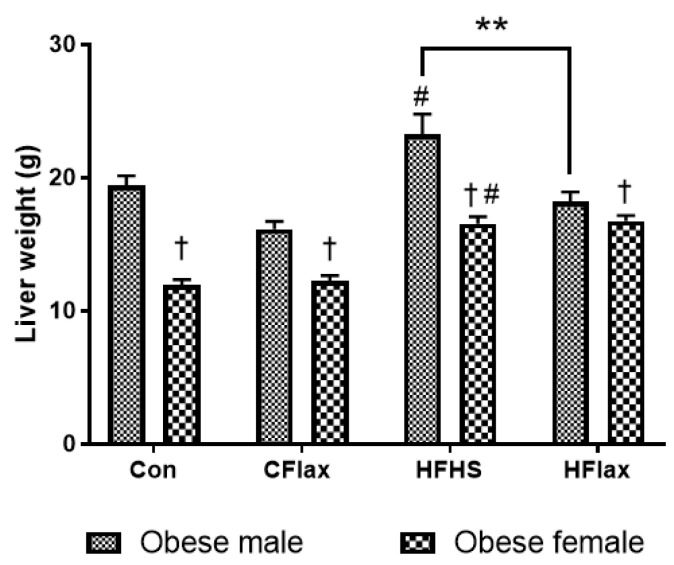
Effect of diet and sex on liver weight. All values presented are mean ± SE. # *p* < 0.05 versus Con diet. ** *p* < 0.01 versus JCR rats on the HFHS diet. Con: control diet; CFlax: flaxseed-supplemented diet; HFHS: high-fat, high-sucrose-supplemented diet; HFlax: high-fat, high-sucrose and flaxseed-supplemented diet; † *p* < 0.05 versus respective male JCR rats on the same diet, two-way ANOVA. N = 8.

Morphological examination revealed increased liver weight in both lean and obese male JCR rats as compared to their respective lean and obese female JCR rats (Table 1). Although diet had no significant effects on liver weights in the lean JCR rats (Table 1), liver weights in obese rats were higher when placed on the HFHS diet (Figure 2). In addition, the male obese rats on a flax-supplemented HFHS diet exhibited lower liver weights than those rats on an HFHS diet alone (Figure 2). Finally, the liver weights were significantly higher in all obese male rats in comparison to obese female rats (Figure 2).

### 3.2. Plasma Levels of Liver Enzymes

**Figure 3:**
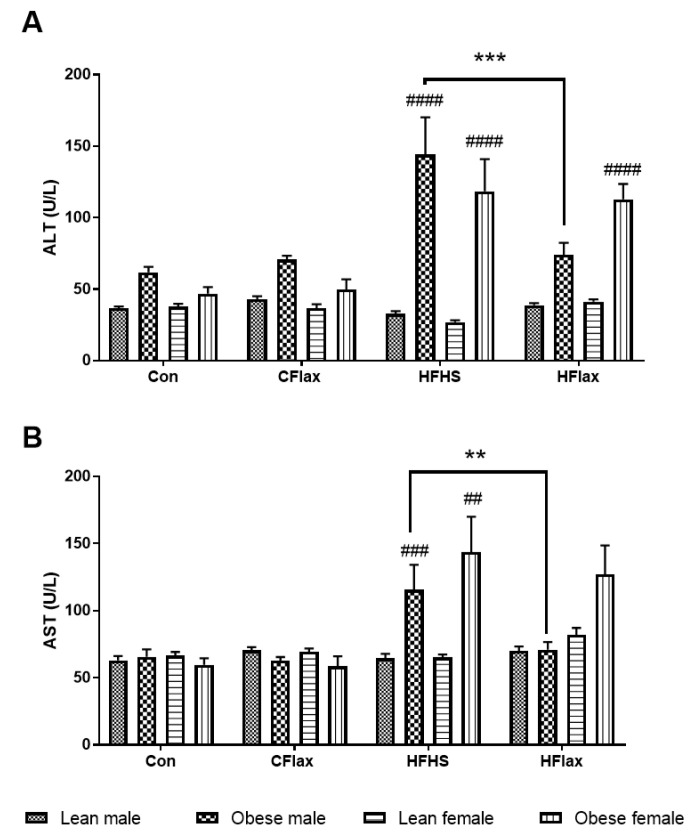
Effect of genotype, sex, and diet on plasma levels of (**A**) alanine aminotransferase (ALT) and (**B**) aspartate aminotransferase (AST). All values presented are mean ± SE. Con: control diet; CFlax: flaxseed-supplemented diet; HFHS: high-fat, high-sucrose-supplemented diet; HFlax: high-fat, high-sucrose and flaxseed-supplemented diet; ## *p* < 0.01, ### *p* < 0.001, and #### *p* < 0.0001 versus respective lean JCR rats on the same diet, two-way ANOVA. ** *p* < 0.01 and *** *p* < 0.001 versus JCR rats on the HFHS diet, two-way ANOVA. N = 8.

The plasma concentrations of enzymes released from the liver were altered as a function of the dietary interventions. Both AST and ALT levels in the circulation were elevated when male and female obese rats were placed on the HFHS diet (Figure 3). When flaxseed was included in the HFHS diet, the levels of both AST and ALT were significantly reduced to normal levels selectively in the obese male rats (Figure 3).

### 3.3. Plasma Lipid Levels

Plasma biochemistry as a result of these interventions has already been reported in detail elsewhere [13]. Plasma total cholesterol concentrations were significantly higher on all four diets in both male and female obese JCR rats when compared to their respective lean controls [13]. Dietary supplementation with flaxseed for 12 weeks significantly lowered both LDL and total cholesterol concentrations in the plasma compared to the respective control groups. HDL cholesterol and triglyceride levels in the plasma were not significantly altered by flaxseed supplementation [13].

A comprehensive list of the observed changes in the plasma fatty acids after 12 weeks of feeding with the different diets is compiled in Supplementary Table S1. Using a two-way ANOVA test with genotype and diet as independent variables revealed significant differences in the plasma levels of myristic acid (C14:0), pentadyclic acid (C15:0), palmitic acid (C16:0), palmitoleic acid (C16:1), heptadecanoic acid (C17:0), cis-10- heptadecanoic acid (C17:1), stearic acid (C18:0), oleic acid (C18:0), cis-vaccenic acid (C18:1n7), linoleic acid (C18:2 *n*-6), gamma-linoleic acid (C18:3 *n*-3), arachidic acid (C20:0), eicosadienoic acid (C20:2 *n*-6), dihomo-gamma-linolenic acid (C20:3 *n*-6), arachidonic acid (C20:4 *n*-6), eicosatrienoic acid (C20:3 *n*-3), behenic acid (C22:0), adrenic acid (C22:0), osbond acid (C22:5 *n*-6), docosapentaenoic acid (C22:5 *n*-3), lignoceric acid (C24:0), and nervonic acid (C24:1). A significant interaction between genotype and diet was observed as obese male JCR rats had significantly higher levels of total saturated fatty acids (SFAs) and lower levels of monounsaturated fatty acids (MUFAs) compared to the lean male JCR rats. Conversely, lean female and obese male JCR rats on the CFlax diet had significantly lower levels of total SFAs and higher levels of MUFA compared to the obese male JCR rats on the Con diet (Supplementary Table S1).

**Figure 4:**
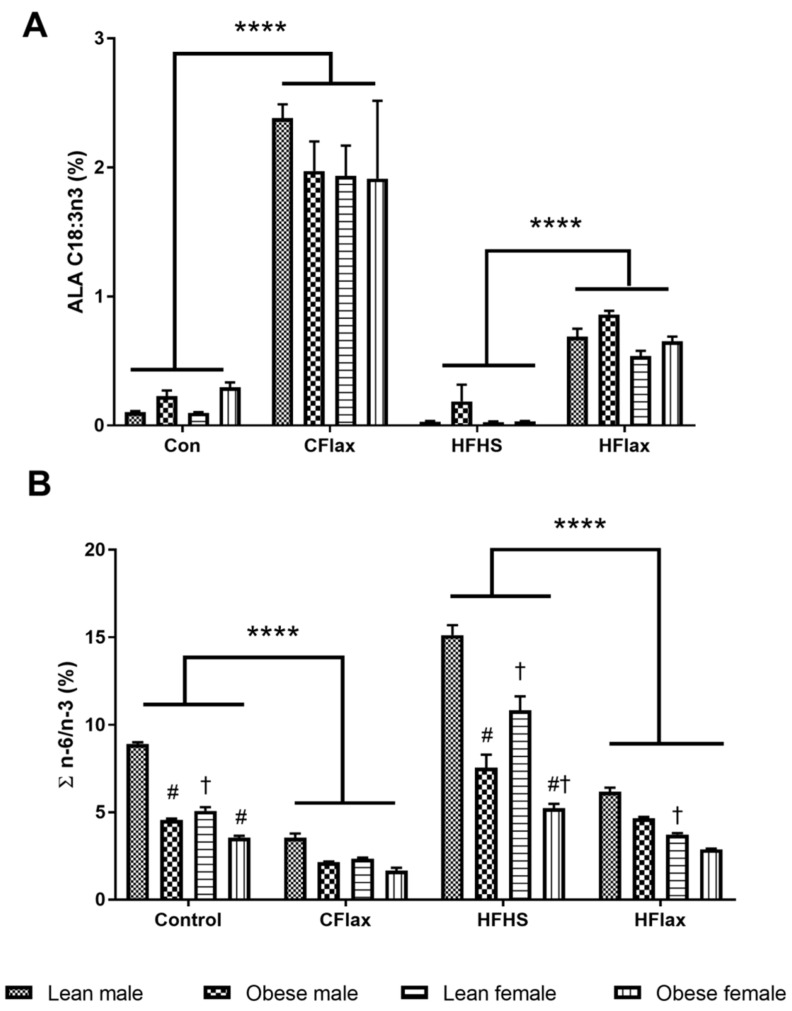
Effect of genotype, sex, and diet on plasma ALA and the ratio of *n*-6 to *n*-3 polyunsaturated fatty acids. All values presented are mean ± SE. (**A**) ALA (C18:3 *n*-3) and (**B**) ratio of *n*-6 and *n*-3 PUFAs (∑*n*-6/*n*-3). CFlax: flaxseed-supplemented diet; HFHS: high-fat, high-sucrose-supplemented diet; HFlax: high-fat, high-sucrose and flaxseed-supplemented diet; **** *p* < 0.0001 versus respective JCR rats on different diets, two-way ANOVA. † *p* < 0.05 versus respective male JCR rats on the same diet, two-way ANOVA. # *p* < 0.05 versus respective lean JCR rats on the same diet, two-way ANOVA. N = 8.

A three-way ANOVA with diet, genotype, and sex as independent variables demonstrated significantly higher levels of the total plasma *n*-3 polyunsaturated fatty acid (PUFAs) in the male and female obese animals on the CFlax diet compared to the obese and lean JCR rats on the Con diet (Supplementary Table S1). Flaxseed supplementation resulted in significantly higher levels of plasma alpha-linolenic acid (ALA, C18:3 *n*-3) in the male and female JCR rats of both genotypes on the CFlax and HFlax diets compared to the Con and HFHS diets, as shown in Figure 4A. A significant increase was also observed in the plasma levels of long-chain *n*-3 PUFAs, EPA (C20:5), in both sexes of lean and obese JCR rats on the CFlax compared to the Con diet. However, no such increase in the levels of EPA was observed in the JCR rats of both sex and genotype on the HFlax diet compared to the HFHS diet (Figure 4). Interestingly, a modest but significant decrease in the levels of another long-chain *n*-3 PUFAs, DHA (C22:6), was noted in lean male and female and obese male JCR rats on the CFlax diet compared to the Con diet, as presented in Figure 4. Conversely, a significant increase in the DHA levels was observed in the lean JCR rats of both sexes and obese male JCR rats on the HFlax compared to the HFHS diet. 

As a result of the increase in the total *n*-3 PUFA levels due to flaxseed supplementation in the diet, a significant lowering of the *n*-6 to *n*-3 ratio was found in all JCR rats on the CFlax and HFlax diets compared to the Con and HFHS diets, respectively (Figure 4B). A strong sex-dependent effect was noted as lean female JCR rats had significantly lower values of *n*-6/*n*-3 ratios compared to the male lean JCR rats on the Con, HFHS, and HFlax diets (Figure 4B).

### 3.4. Liver Fatty Acid Profile

A comprehensive depiction of the observed changes in the liver fatty acids after 12 weeks of feeding with the different diets is compiled in Supplementary Figures S1–S4. For the sake of clarity in presentation and statistical analysis, the 26 different fatty acid species identified in the analysis were separated according to their characteristics (saturated, monounsaturated, polyunsaturated, and *n*-3 polyunsaturated) and only the statistically significant differences versus the respective control group, or HFlax versus the respective HFHS group, or obese vs. lean within the same respective dietary group are shown (Supplementary Figures S1–S4) (* *p* < 0.05).

A clear trend in statistical significance was observed in the saturated fatty acid levels between male and female animals. Almost without exception in all groups, no matter the dietary intervention, the concentration of saturated fatty acids in obese females was significantly lower than the obese males (Supplementary Figure S1) (* *p* < 0.05). This effect of sex in the obese group was also exhibited in the monounsaturated fatty acids (Supplementary Figure S2) (* *p* < 0.05).

Supplementation of the HFHS diet with flaxseed resulted in a significant decrease in selected monounsaturated fatty acids (14:1, 16:1, 18:1n7c, and 20:1) in the liver in comparison to the HFHS diet alone (Supplementary Figure S2) (* *p* < 0.05).

In the polyunsaturated fatty acids, again a general trend in lower values in the female obese animals in comparison to the male obese rats was observed (Supplementary Figure S3) (* *p* < 0.05). However, flaxseed increased a number of polyunsaturated fatty acids in the liver tissue of both flax-supplemented diets and HFHS-flax-supplemented diets (Supplementary Figure S3) (* *p* < 0.05).

**Figure 5:**
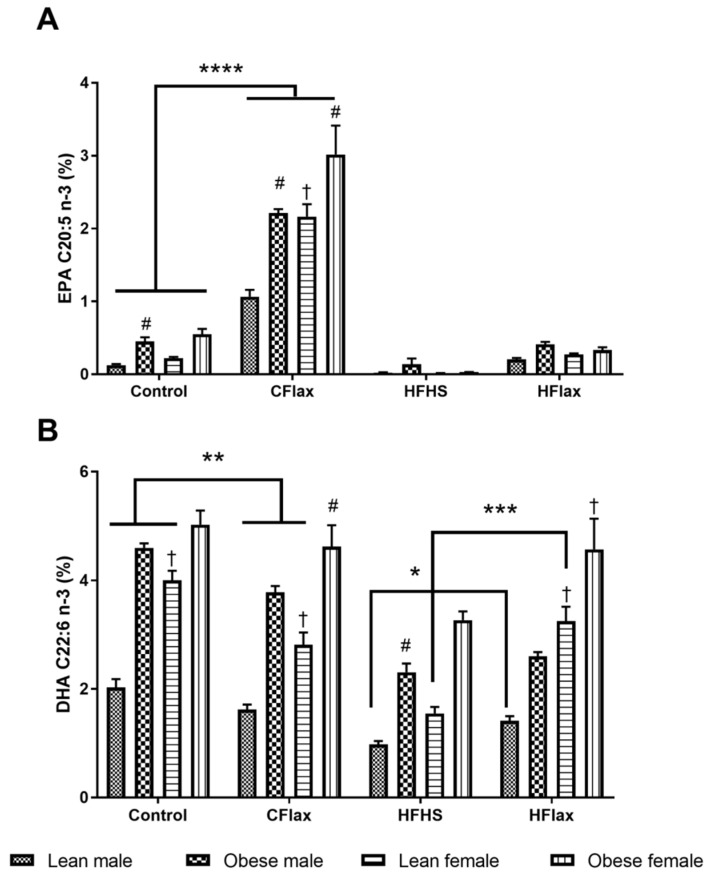
Effect of genotype, sex, and diet on plasma EPA and DHA. All values presented are mean ± SE. (**A**) EPA (C20:5 *n*-3) and (**B**) DHA (C22:6 *n*-3). CFlax: flaxseed-supplemented diet; HFHS: high-fat, high-sucrose-supplemented diet; HFlax: high-fat, high-sucrose and flaxseed-supplemented diet; **** *p* < 0.0001, *** *p* < 0.001, ** *p* < 0.01, and * *p* < 0.05 versus respective JCR rats on different diets, two-way ANOVA. † *p* < 0.05 versus respective male JCR rats on the same diet, two-way ANOVA. # *p* < 0.05 versus respective lean JCR rats on the same diet, two-way ANOVA. N = 8.

This general trend for an increase in fatty acid levels in the flaxseed-supplemented groups was also exhibited in *n*-3 polyunsaturated fatty acid lipids (Figure 5) (* *p* < 0.05). This was particularly evident in the liver 18-3n3 fatty acid levels, undoubtedly because of incorporation of this fatty acid from the rich content of this fatty acid in flaxseed which was presented in the diet.

### 3.5. Liver Cholesterol and Triglyceride Content

**Figure 6:**
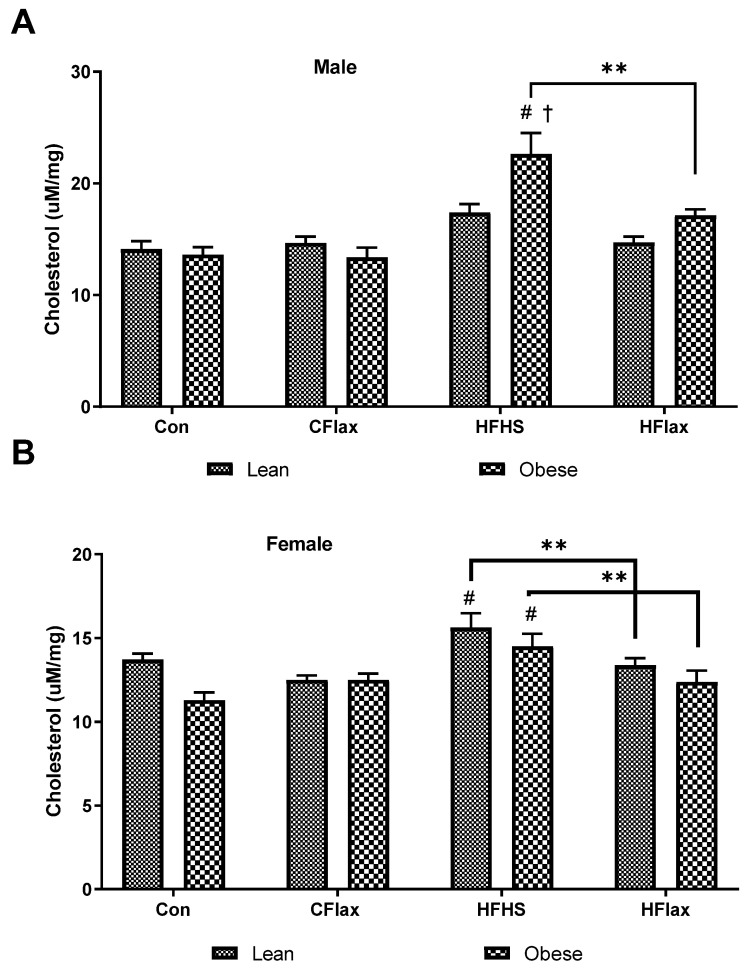
Hepatic total cholesterol as a function of the dietary interventions in lean and obese male and female JCR:LA-cp rats. All values presented are mean ± SE. (**A**) Hepatic cholesterol in male animals and (**B**) hepatic cholesterol in female animals. CFlax: flaxseed-supplemented diet; HFHS: high-fat, high-sucrose-supplemented diet; HFlax: high-fat, high-sucrose and flaxseed-supplemented diet; ** *p* < 0.01 versus respective JCR rats on different diets, two-way ANOVA. † *p* < 0.05 versus respective JCR rats on the same diet, two-way ANOVA. # *p* < 0.05 versus JCR rats on the control diet, two-way ANOVA. N = 8.

Total cholesterol content in the livers of male JCR:LA rats was relatively constant until the obese animals were fed an HFHS diet (Figure 6A). Cholesterol content was significantly increased in the livers from the HFHS rats (Figure 6A). The addition of flaxseed to the HFHS diet significantly decreased the cholesterol levels in the livers from these animals (Figure 6A). In the female animals, the HFHS diet increased the liver cholesterol content in both lean and obese animals and the addition of flaxseed to the HFHS diet decreased the cholesterol levels in both lean and obese rats (Figure 6B).

**Figure 7:**
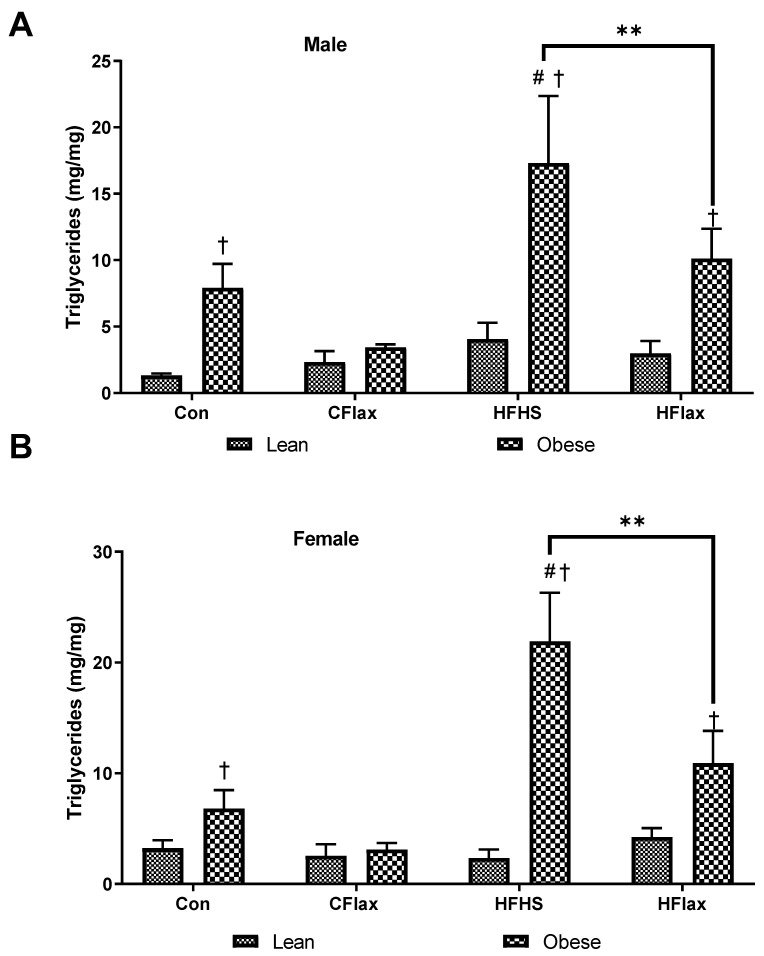
Hepatic triglycerides as a function of the dietary interventions in lean and obese male and female JCR:LA-cp rats. All values presented are mean ± SE. (**A**) Hepatic trigyclerides in male animals and (**B**) hepatic triglycerides in female animals. CFlax: flaxseed-supplemented diet; HFHS: high-fat, high-sucrose-supplemented diet; HFlax: high-fat, high-sucrose and flaxseed-supplemented diet; ** *p* < 0.01 versus respective JCR rats on different diets, two-way ANOVA. † *p* < 0.05 versus respective JCR rats on the same diet, two-way ANOVA. # *p* < 0.05 versus JCR rats on the control diet, two-way ANOVA. N = 8.

Triglyceride levels were measured in the livers of the JCR:LA rats (Figure 7). Obese male rats exhibited an elevated liver triglyceride content in comparison to the lean controls on the same chow diet (Figure 7A). Animals on an HFHS diet showed a significant increase in triglyceride content and this was reduced by the inclusion of flaxseed in the HFHS diet (Figure 7A). This same qualitative response as was exhibited by the male rats was shown by the female animals (Figure 7B).

### 3.6. Lipid Metabolism Protein Expression in Liver

Protein expression of key proteins involved in lipid metabolism in the liver of the JCR:LA rats was measured by western blots. No significant changes in the expression of SREBP-1, HMGCR, CYP7A1, and CD36 were observed in the liver as a function of diet, sex, or genetic predisposition.

### 3.7. Correlative Analysis of AST and ALT Changes with Lipid Levels

**Figure 8:**
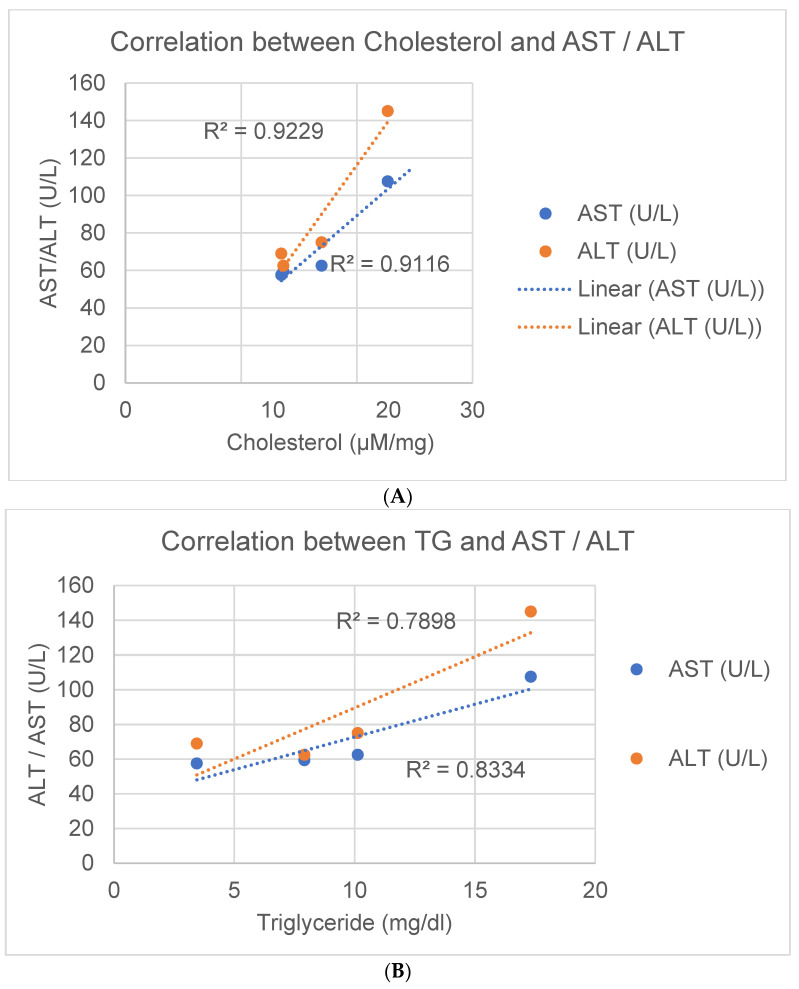
Linear correlation of hepatic cholesterol (**A**) and hepatic triglycerides (**B**) with AST (green line and dots) and ALT (red line and dots) release into the plasma.

The release of the liver proteins AST and ALT into the plasma of the JCR:LA rats was correlated with hepatic concentrations of cholesterol and triglycerides (Figure 8), and polyunsaturated fatty acids (Supplementary Figure S5). There was a significant positive correlation between the release of both AST and ALT into the plasma with liver cholesterol (Figure 8A) and liver triglycerides (Figure 8B). Conversely, moderate (Supplementary Figure S5A—C18:3n3) to strong (Supplementary Figure S5B—C22:6n3 and Supplementary Figure S5C—C22:5n3) negative logarithmic correlations were observed for these polyunsaturated *n*-3 fatty acids with both ALT and AST (Supplementary Figure S5).

### 3.8. Principal Component Analysis for the Biochemical Parameters

**Figure 9:**
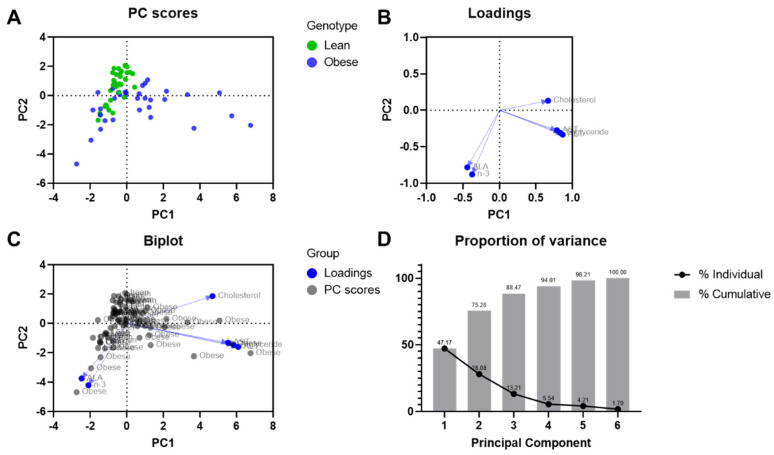
Principal component analysis on biochemical parameters. (**A**) Scores plot; (**B**) loadings plot; (**C**) biplot; and (**D**) proportion of variance plot.

Figure 9 shows the result of the examination of six biochemical variables in lean and obese rats of both sexes, on four different diets, by principal component analysis. ALA and *n*-3 are mainly projected on principal component 1, while lipid and liver enzymes levels have a strong influence on principal component 2. This suggests that the higher the ALA levels, the lower the cholesterol, TGs, and liver enzyme levels. The eigenvalues of the first two principal components were above zero. The 47.17% of variance was explained by the first principal component and the 28.08% of variance by the second component.

## 4. Discussion

The JCR:LA-cp rat is an appropriate animal model to represent NAFLD as shown by previous research [8] and in the JCR:LA-cp animals under the experimental conditions used in the present study. This is supported by the increase in liver weight when the animals were placed on an HFHS diet, the release of AST and ALT into the plasma from the liver when administered the HFHS diet, and the increase in liver lipid levels (cholesterol, triglycerides, saturated fats). Although both male and female animals showed characteristics of NAFLD, it was more evident in the present study in the male obese animals on an HFHS diet. This is consistent with the human data which have shown an increased prevalence in males [2,6,7].

Dietary flaxseed has been shown to induce cardioprotective effects in both experimental animal studies [13,15,17,20] and human clinical trials [21]. It was hypothesized in the present study that dietary supplementation with flaxseed may also protect against liver disease. The JCR:LA-cp model of NAFLD has been shown previously to be sensitive to dietary interventions [22,23,24,25,26]. The data produced here would support the contention that supplementation of the diet with flaxseed has a significant protective action in this model of NAFLD. This is particularly evident in the male obese animals. This conclusion is supported by the inhibition of the release of liver proteins AST and ALT, as well as an improvement in liver weight and lipid content (triglycerides, cholesterol, fatty acids). Based on the PCA, it was determined that ALA and *n*-3 were positively correlated. There was a minor difference between liver enzymes and TG levels; however, a stronger effect was related to cholesterol levels. Reduction in plasma cholesterol levels as a result of dietary flaxseed is in agreement with PCA results. This biochemical profile and the change in these biomarkers argue strongly that dietary flaxseed induced a positive change in hepatic health.

The mechanism for this significant change in liver health is unclear but several possibilities can be considered or ruled out based on the data obtained in this study. Flaxseed did not alter the expression of proteins associated with lipid metabolism in the liver. However, the composition of the lipids within the liver tissue did change. Saturated fats decreased and polyunsaturated fats increased in the liver tissue when flaxseed was introduced into the diet even in the presence of the HFHS components. This is consistent with previous study of the incorporation of omega-3 fatty acids from a flaxseed-supplemented diet into tissues of the body [27]. This correlated with the inhibited release of the liver proteins AST and ALT into the circulation (Supplementary Figure S5). The tissue cholesterol and triglyceride levels in the liver were also strongly associated with the lowering of these biomarkers of liver damage (Figure 7). Flaxseed is known to lower circulating levels of cholesterol in clinical trials, presumably via the large fibre load delivered by the flaxseed [17]. Based on these results, it is reasonable to conclude that the inclusion of flaxseed in the diet can alter both the amounts and types of detrimental hepatic lipids during fatty liver disease. The anti-inflammatory and anti-oxidative actions [13] of the rich content of polyunsaturated fatty acids and lignans found in flaxseed would also potentially contribute to the health benefits of dietary flaxseed.

## 5. Conclusions

The JCR:LA-cp rats mimic NAFLD by exhibiting elevated plasma levels of AST and ALT along with higher levels of lipid within the liver. As this rat strain exhibits obesity, high lipid levels, hypertension, and insulin resistance, it could also be useful for the study of metabolic dysfunction-associated fatty liver disease (MAFLD) [28], a newly adopted term that has shown superiority over NAFLD in identifying high-risk patients and predicting overall mortality. The inclusion of ground flaxseed into the diet was beneficial to liver health even in the presence of an overall diet that was enriched in fat and sucrose, as evident by improved liver weight, lowered tissue lipid content, and reduced plasma release of AST and ALT. Flaxseed favorably altered liver lipid composition and this change correlated with reduced ALT, AST, and lower hepatic cholesterol/triglyceride levels, indicating decreased liver damage. Further studies may be warranted to dissect molecular mechanisms and to explore different chemical components of flaxseed. Ultimately, clinical studies with NAFLD may be required to determine if supplementation of the diet with ground flaxseed can provide some relief or a reversal of the hepatic injury associated with this prevalent disease condition in the human population.

## Figures and Tables

**Table 1:** Liver weight of JCR:LA lean animals.

Measure	Genotype	Sex	Diet			
			Control	CFlax	HFHS	HFlax
Liver weight, g	Lean	Male	8.69 ± 0.25	8.62 ± 0.19	8.17 ± 0.26	8.12 ± 0.32
		Female	6.17 ± 0.36	6.49 ± 0.40	5.10 ± 0.16	5.39 ± 0.20

Values are presented as mean ± SE (*n* = 8). The liver weights were calculated as a ratio of dry/wet weights. No values differed significantly as a function of dietary intervention (*p* > 0.05). All values were significantly different from each respective group as a function of sex (*p* < 0.05). CFlax: 10% ground flaxseed-supplemented control diet; HFHS: high-fat, high-sucrose diet; HFlax: 10% ground flaxseed-supplemented high-fat/high-sucrose diet.

## Data Availability

All data have been archived in publicly accessible digital sites at the Albrechtsen Research Centre within St Boniface Hospital, Winnipeg, Canada.

## Supplementary Materials

The following supporting information can be downloaded at: https://www.mdpi.com/article/10.3390/nu16040466/s1, Figure S1: Representative chromatograms obtained from a JCR:LA-*cp* lean animal and a standard; Figure S2: Composition of liver saturated fatty acids in lean and obese male and female JCR:LA rats as a function of diet; Figure S3: Composition of liver monounsaturated fatty acids in lean and obese male and female JCR:LA rats as a function of diet.; Figure S4: Composition of liver polyunsaturated fatty acids in lean and obese male and female JCR:LA rats as a function of diet; Figure S5: Composition of liver *n*-3 polyunsaturated fatty acids in lean and obese male and female JCR:LA rats as a function of diet; Figure S6: Logarithmic correlation between polyunsaturated fatty acids and ALT and AST release into the plasma; Table S1: Circulating fatty acid analysis.

## Abbreviations

ALT	Alanine aminotransferase
ALA	Alpha-linolenic acid
AST	aspartate aminotransferase
HFHS	high-fat, high-sucrose diet
NAFLD	Non-alcoholic fatty liver disease
