# Supplementary material for: Beneficial Effects of Dietary Flaxseed on Non-Alcoholic Fatty Liver Disease
Source: Nutrients. 2024 Feb 6;16(4):466. doi: 10.3390/nu16040466 (PMC10892801; doi:10.3390/nu16040466)
Supplement: Supplementary file 1 [file nutrients-16-00466-s001.zip › nutrients-2746560-supplementary.pdf]

Table S1. Circulating fatty acid analysis.

Fatty acids	Genotype	Sex	Diet			
			Con	CFlax	HFHS	HFlax
C14:0 (Myristic acid)	Lean	Male	0.08±0.01[a]	0.10±0.01[a]	0.12±0.02[a]	0.12±0.02[a]
		Female	0.09±0.01[a]	0.08±0.01[a]	0.10±0.00[a]	0.08±0.01[a]
	Obese	Male	0.24±0.03[a****]	0.20±0.02[a*]	0.21±0.03[a]	0.17±0.02[a]
		Female	0.20±0.02[a***]	0.15±0.02[a,b]	0.12±0.01[b]ǂ	0.13±0.01[b]
C15:0 (Pentadyclic acid)	Lean	Male	0.13±0.01[a]	0.15±0.01[a]	0.06±0.01[b]	0.07±0.00 [b]
		Female	0.09±0.01[a]ǂ	0.09±0.00[a]ǂ	0.05±0.00[b]	0.05±0.00[b]
	Obese	Male	0.13±0.01[a]	0.11±0.01[a*]	0.07±0.00[b]	0.06±0.00[b]
		Female	0.09±0.00[a]ǂ	0.09±0.01[a]	0.05±0.00[b]	0.05±0.00[b]
C16:0	Lean	Male	11.25±0.47[a]	10.35±0.39[a]	11.66±0.48[a]	11.35±0.41[a]

(Palmitic acid)		Female	10.28±0.38^{a}	8.82±0.15^{b}	9.65±0.19^{a,b}	9.47±0.33^{a,b}
	Obese	Male	17.69±0.63^{a****}	15.65±0.18^{b,c****}	15.90±0.33^{b****}	14.20±0.14^{c**}
		Female	13.42±0.79^{a*}ǂ	11.81±1.33^{a}ǂ	10.73±0.26^{a}ǂ	10.91±0.83^{a}ǂ
C16:1<br><br>(Palmitoleic acid)	Lean	Male	0.09±0.01^{a}	0.11±0.01^{a}	0.13±0.01^{a}	0.09±0.01^{a}
		Female	0.18±0.01^{a,c}	0.24±0.02^{b}	0.24±0.01^{b,c}	0.22±0.01^{b,c}
	Obese	Male	1.06±0.12^{a****}	1.18±0.14^{a****}	0.75±0.09^{a***}	0.66±0.12^{a**}
		Female	1.12±0.13^{a****}	0.97±0.19^{a,b***}	0.49±0.05^{b}	0.45±0.10^{b}
C17:0<br><br>(Heptadecanoic acid)	Lean	Male	0.34±0.03^{a}	0.34±0.01^{a}	0.16±0.01^{b}	0.14±0.01^{b}
		Female	0.32±0.03^{a}	0.34±0.03^{a}	0.14±0.01^{b}	0.14±0.01^{b}
	Obese	Male	0.24±0.02^{a**}	0.22±0.02^{a,b***}	0.17±0.01^{b}	0.18±0.01^{b}
		Female	0.22±0.01^{a**}	0.22±0.02^{a**}	0.13±0.00^{b}	0.14±0.01^{b}
C17:1	Lean	Male	48.19±3.20^{a}	49.22±1.94^{a}	44.89±1.89^{a}	47.72±2.16^{a}

(*cis*-10-Heptadecanoic acid)		Female	40.77±2.28[a]	47.33±1.45[a]	46.23±0.78[a]	44.80±2.35[a]
	Obese	Male	24.91±1.99[a****]	33.50±1.69[b***]	31.93±1.50[b**]	34.23±1.01[b**]
		Female	30.64±3.53[a]	33.82±5.73[a]	44.11±1.75[a]	40.25±4.34[a]
C18:0 (Stearic acid)	Lean	Male	9.52±0.68[a]	9.52±0.37[a]	12.31±0.63[b]	11.62±0.35[b]
		Female	15.56±0.69[a]ǂ	13.35±0.38[b]ǂ	14.79±0.31[a,b]	15.21±0.56[a,b]ǂ
	Obese	Male	12.57±0.21[a**]	11.55±0.37[a]	13.40±0.69[a]	13.70±0.22[a]
		Female	15.04±0.54[a]	15.20±0.64[a]ǂ	14.43±0.44[a]	15.55±1.23[a]
C18:1 (Oleic acid)	Lean	Male	3.22±0.19[a]	3.28±0.19[a]	6.21±0.11[b]	5.90±0.31[b]
		Female	2.72±0.10[a]	2.43±0.05[a]	4.91±0.11[b]	4.55±0.24[b]
	Obese	Male	7.55±0.69[a****]	5.73±0.28[b***]	8.38±0.29[a**]	7.07±0.23[a,b]
		Female	8.22±0.98[a****]	6.38±1.32[a**]	5.62±0.35[a]ǂ	5.75±0.25[a]
	Lean	Male	0.89±0.06[a,b]	0.95±0.04[a]	0.77±0.01[b]	0.74±0.03[b]

C18:1n7 (*cis*-Vaccenic acid)		Female	0.70±0.08[a]	0.83±0.11[a]	0.61±0.01[a]	0.68±0.10[a]
	Obese	Male	1.39±0.02[a]	1.18±0.05[a,b]	0.90±0.02[b]	1.07±0.24[a,b]
		Female	0.88±0.06[a]ǂ	0.84±0.10[a]ǂ	0.69±0.03[a]	0.70±0.04[a]
C18:2 n-6 (Linoleic acid)	Lean	Male	9.87±0.40[a]	12.26±0.51[b]	7.26±0.22[c]	7.92±0.28[c]
		Female	8.73±0.43[a]	9.09±0.33[a]ǂ	5.71±0.53[b]	5.87±0.24[b]
	Obese	Male	13.56±1.00[a***]	11.33±0.53[a,b]	9.35±0.31[b,c]	8.51±0.23[c]
		Female	9.99±0.76[a]ǂ	8.70±0.79[a]ǂ	4.36±0.19[b]ǂ	5.48±0.56[b]ǂ
C18:3 n-6 (Gamma-linolenic acid)	Lean	Male	0.03±0.01[a]	0.03±0.00[a]	0.03±0.01[a]	0.02±0.01[a]
		Female	0.05±0.01[a]	0.04±0.00[a]	0.05±0.01[a]	0.05±0.02[a]
	Obese	Male	0.07±0.00[a**]	0.05±0.01[b]	0.04±0.00[b]	0.04±0.01[b]
		Female	0.09±0.02[a]	0.06±0.01[a,b]	0.04±0.00[b]	0.03±0.00[b]
C20:0	Lean	Male	0.05±0.02[a]	0.05±0.00[a]	0.07±0.01[a]	0.07±0.01[a]

(Arachidic acid)		Female	0.05±0.00[a]	0.06±0.01[a]	0.08±0.01[a]	0.07±0.01[a]
	Obese	Male	0.05±0.00[a]	0.05±0.01[a]	0.06±0.01[a]	0.06±0.00[a]
		Female	0.04±0.00[a]	0.03±0.00[a]	0.04±0.00[a**]	0.04±0.00[a*]
C20:1<br><br>(Eicosenoic acid)	Lean	Male	0.19±0.05[a]	0.19±0.02[a]	0.19±0.02[a]	0.18±0.01[a]
		Female	0.19±0.07[a]	0.25±0.13[a]	0.14±0.00[a]	0.28±0.13[a]
	Obese	Male	0.14±0.02[a]	0.11±0.03[a]	0.16±0.01[a]	0.09±0.03[a]
		Female	0.11±0.01[a]	0.09±0.01[a]	0.12±0.01[a]	0.21±0.11[a]
C20:2 n-6<br><br>(Eicosadienoic acid)	Lean	Male	0.22±0.01[a]	0.14±0.01[b]	0.08±0.00[c]	0.07±0.01[c]
		Female	0.13±0.01[aǂ]	0.09±0.01[a,bǂ]	0.08±0.01[b]	0.08±0.01[b]
	Obese	Male	0.25±0.01[a]	0.17±0.01[b]	0.20±0.01[c]	0.17±0.01[b]
		Female	0.13±0.02[aǂ]	0.10±0.01[a,bǂ]	0.08±0.00[bǂ]	0.10±0.01[a,bǂ]
C20:3 n-6	Lean	Male	0.15±0.01[a,c]	0.33±0.01[b]	0.20±0.01[c]	0.20±0.02[c]

(Dihomo-gamma-linolenic acid)		Female	0.20±0.02[a]	0.31±0.02[a]	0.26±0.03[a]	0.26±0.04[a]
	Obese	Male	1.56±0.05[a]****	1.13±0.05[b,c]****	1.38±0.09[a,b]****	1.00±0.06[c]****
		Female	0.74±0.08[a]****ǂ	0.66±0.03[a]****ǂ	0.34±0.03[b]ǂ	0.51±0.05[a,b]**ǂ
C20:4 n-6 (Arachidonic acid)	Lean	Male	12.68±1.30[a]	6.74±0.44[b]	13.23±0.57[a]	10.14±0.53[a]
		Female	14.37±0.84[a]	8.31±0.36[b]	13.17±0.65[a]	12.39±1.20[a]
	Obese	Male	11.95±0.47[a]	7.73±0.29[b]	12.75±0.73[a]	12.88±0.37[a]
		Female	11.95±0.33[a]	8.90±0.72[b]	13.37±0.52[c]	12.56±1.30[a]
C20:3 n-3 (Eicosatrienoic acid)	Lean	Male	0.09±0.07[a]	0.07±0.01[a]	0.15±0.03[a]	0.20±0.05[a]
		Female	0.02±0.01[a]	0.06±0.01[a]	0.12±0.01[b]	0.17±0.02[b]
	Obese	Male	0.02±0.00[a]	0.08±0.01[a]	0.37±0.05[b]**	0.32±0.05[b]
		Female	0.01±0.00[a]	0.08±0.01[b]	0.11±0.01[b,c]ǂ	0.14±0.01[c]
C22:0	Lean	Male	0.10±0.02[a]	0.09±0.01[a]	0.13±0.02[a]	0.14±0.01[a]

(Behenic acid)		Female	0.16±0.01[a]	0.13±0.00[a]	0.16±0.01[a]	0.21±0.02[b]ǂ
	Obese	Male	0.10±0.00[a]	0.12±0.00[a,b]	0.13±0.01[a,b]	0.16±0.02[b]
		Female	0.08±0.00[a*]	0.08±0.01[a]	0.12±0.00[a]	0.12±0.03[a***]
C22:4<br><br>(Adrenic acid)	Lean	Male	0.13±0.02[a]	0.05±0.00[b]	0.20±0.01[c]	0.08±0.02[a,b]
		Female	0.14±0.01[a]	0.04±0.01[b]	0.30±0.02[c]ǂ	0.10±0.02[a,b]
	Obese	Male	0.15±0.01[a]	0.07±0.01[b]	0.14±0.02[a]	0.15±0.02[a]
		Female	0.17±0.02[a]	0.06±0.01[b]	0.32±0.02[c]ǂ	0.14±0.02[a]
C22:5 n-6<br><br>(Osbond acid)	Lean	Male	0.03±0.01[a]	0.03±0.00[a]	0.19±0.02[a]	0.03±0.01[a]
		Female	0.06±0.00[a]	0.01±0.00[a]	0.82±0.05[c]ǂ	0.06±0.02[a]
	Obese	Male	0.07±0.01[a]	0.06±0.01[a]	0.09±0.02[a***]	0.04±0.00[a]
		Female	0.06±0.01[a]	0.02±0.00[a]	0.67±0.09[b]ǂ	0.06±0.02[a]
C22:5 n-3	Lean	Male	0.29±0.01[a]	0.87±0.09[b]	0.23±0.02[a]	0.58±0.04[c]

(Docosapentaenoic acid)		Female	0.34±0.03[a]	0.73±0.06[b]	0.20±0.02[a]	0.82±0.05[b,c]
	Obese	Male	0.78±0.05[a***]	1.57±0.02[b****]	0.34±0.10[c]	0.81±0.02[a]
		Female	0.68±0.07[a]	1.42±0.23[a***]	0.25±0.01[b]	0.86±0.06[a]
C24:0 (Lignoceric acid)	Lean	Male	0.16±0.06[a]	0.21±0.03[a]	0.17±0.02[a]	0.16±0.02[a]
		Female	0.32±0.04[a]ǂ	0.34±0.03[a]ǂ	0.16±0.01[b]	0.24±0.03[a,b]
	Obese	Male	0.18±0.02[a,b]	0.21±0.02[a]	0.13±0.01[b]	0.21±0.03[a]
		Female	0.12±0.02[a***]	0.12±0.01[a****]	0.08±0.01[a]	0.12±0.02[a*]
C24:1 (Nervonic acid)	Lean	Male	0.17±0.06[a]	0.16±0.03[a]	0.21±0.03[a]	0.24±0.02[a]
		Female	0.35±0.01[a]ǂ	0.30±0.01[a]	0.29±0.02[a]	0.30±0.04[a]
	Obese	Male	0.20±0.01[a]	0.20±0.02[a]	0.33±0.02[b*]	0.31±0.00[b]
		Female	0.13±0.01[a***]	0.13±0.01[a*]	0.26±0.00[a]	0.24±0.07[a]
∑SFA	Lean	Male	21.60±1.21[a]	20.77±0.76[a]	24.68±1.17[a]	23.61±0.80[a]

		Female	26.86±1.00[a]	23.21±0.54[b]	25.12±0.45[a,b]	25.47±0.93[a,b]
	Obese	Male	31.19±0.63[a****]	28.10±0.54[b****]	30.08±0.66[a,b**]	28.74±0.36[a,b**]
		Female	29.22±1.29[a]	27.70±1.87[a]	25.70±0.68[a]	27.05±2.07[a]
∑MUFA	Lean	Male	52.70±3.00[a]	53.88±1.57[a]	52.70±1.76[a]	54.93±1.71[a]
		Female	44.79±2.28[a]	51.22±1.33[a,b]	52.56±0.81[b]	50.71±2.30[a,b]
	Obese	Male	35.10±1.57[a****]	41.75±1.12[b**]	42.61±1.71[b**]	43.58±0.76[b**]
		Female	41.07±2.67[a]	42.18±4.44[a]	51.41±1.40[a]	47.56±4.45[a]
∑n-6	Lean	Male	23.13±1.66[a]	19.66±0.48[a,b]	21.18±0.54[a,b]	18.42±0.81[b]
		Female	23.66±1.19[a]	17.87±0.59[b]	20.39±0.32[a,b]	18.75±1.07[b]
	Obese	Male	27.62±0.79[a]	20.52±0.28[b]	23.95±0.78[c]	22.77±0.30[b,c]
		Female	23.14±1.04[a]	18.48±1.09[a]	19.18±0.68[a]ǂ	18.86±1.83[a]
∑n-3	Lean	Male	2.60±0.18[a,c]	5.67±0.43[b]	1.41±0.07[a]	3.01±0.18[c]

		Female	4.67±0.20^{a}ǂ	7.70±0.31^{b}ǂ	1.91±0.14^{c}	5.05±0.31^{a}ǂ
	Obese	Male	6.07±0.19^{a****}	9.62±0.32^{b****}	3.34±0.47^{c**}	4.90±0.12^{a**}
		Female	6.56±0.40^{a}	11.62±1.63^{b**}	3.69±0.17^{a}	6.53±0.55^{a}
∑PUFA	Lean	Male	25.72±1.84^{a}	25.33±0.85^{a}	22.59±0.58^{a}	21.43±0.96^{a}
		Female	28.33±1.30^{a}	25.56±0.83^{a,b}	22.30±0.39^{b}	23.80±1.38^{b}
	Obese	Male	33.69±0.95^{a***}	30.14±0.59^{a,b}	27.29±1.07^{b}	27.67±0.40^{b*}
		Female	29.70±1.41^{a}	30.10±2.59^{a}	22.88±0.73^{a}	25.38±2.38^{a}

Values are presented as mean ± SE. Row means without a common superscript letter (a, b, and c) differ significantly, $P<0.05$.

****$P<0.0001$ ***$P<0.001$ **$P<0.01$ *$P<0.05$ versus respective lean JCR rats on the same diet, 2-way ANOVA.

ǂ $P<0.05$ versus respective male JCR rats on the same diet, 2-way ANOVA.

N=8. Con, control diet; CFlax, 10% ground flaxseed supplemented control diet; HFHS, high-fat high sucrose diet; HFlax, 10% ground flaxseed supplemented high-fat high sucrose diet.

Figure Legends

Figure S1. Composition of liver saturated fatty acids in lean and obese male and female JCR:LA rats as a function of diet. Abbreviations: CTR: control diet; FX: flaxseed supplemented diet; HFHS: high fat and high sucrose supplemented diet; HFX: high fat, high sucrose and flaxseed supplemented diet; *P< 0.05 versus respective control group; **P< 0.05 versus respective HFHS group; ***P<0.05 obese vs lean within same respective dietary group; (n=7 or 8).

Figure S2. Composition of liver monounsaturated fatty acids in lean and obese male and female JCR:LA rats as a function of diet. Abbreviations: CTR: control diet; FX: flaxseed supplemented diet; HFHS: high fat and high sucrose supplemented diet; HFX: high fat, high sucrose and flaxseed supplemented diet; *P< 0.05 versus respective control group; **P< 0.05 versus respective HFHS group; ***P<0.05 obese vs lean within same respective dietary group; (n=7 or 8).

Figure S3. Composition of liver polyunsaturated fatty acids in lean and obese male and female JCR:LA rats as a function of diet. Abbreviations: CTR: control diet; FX: flaxseed supplemented diet; HFHS: high fat and high sucrose supplemented diet; HFX: high fat, high sucrose and flaxseed supplemented diet; *P< 0.05 versus respective control group; **P< 0.05 versus respective HFHS group; ***P<0.05 obese vs lean within same respective dietary group; (n=7 or 8).

Figure S4. Composition of liver n-3 polyunsaturated fatty acids in lean and obese male and female JCR:LA rats as a function of diet. Abbreviations: CTR: control diet; FX: flaxseed supplemented diet; HFHS: high fat and high sucrose supplemented diet; HFX: high fat, high sucrose and flaxseed supplemented diet; *P< 0.05 versus respective control group; **P< 0.05 versus respective HFHS group; ***P<0.05 obese vs lean within same respective dietary group; (n=7 or 8).

Figure S5. Logarithmic correlation between polyunsaturated fatty acids and ALT and AST release into the plasma. C18:3n3 (A); C22:6n3 (B); C22:5n3 (C).

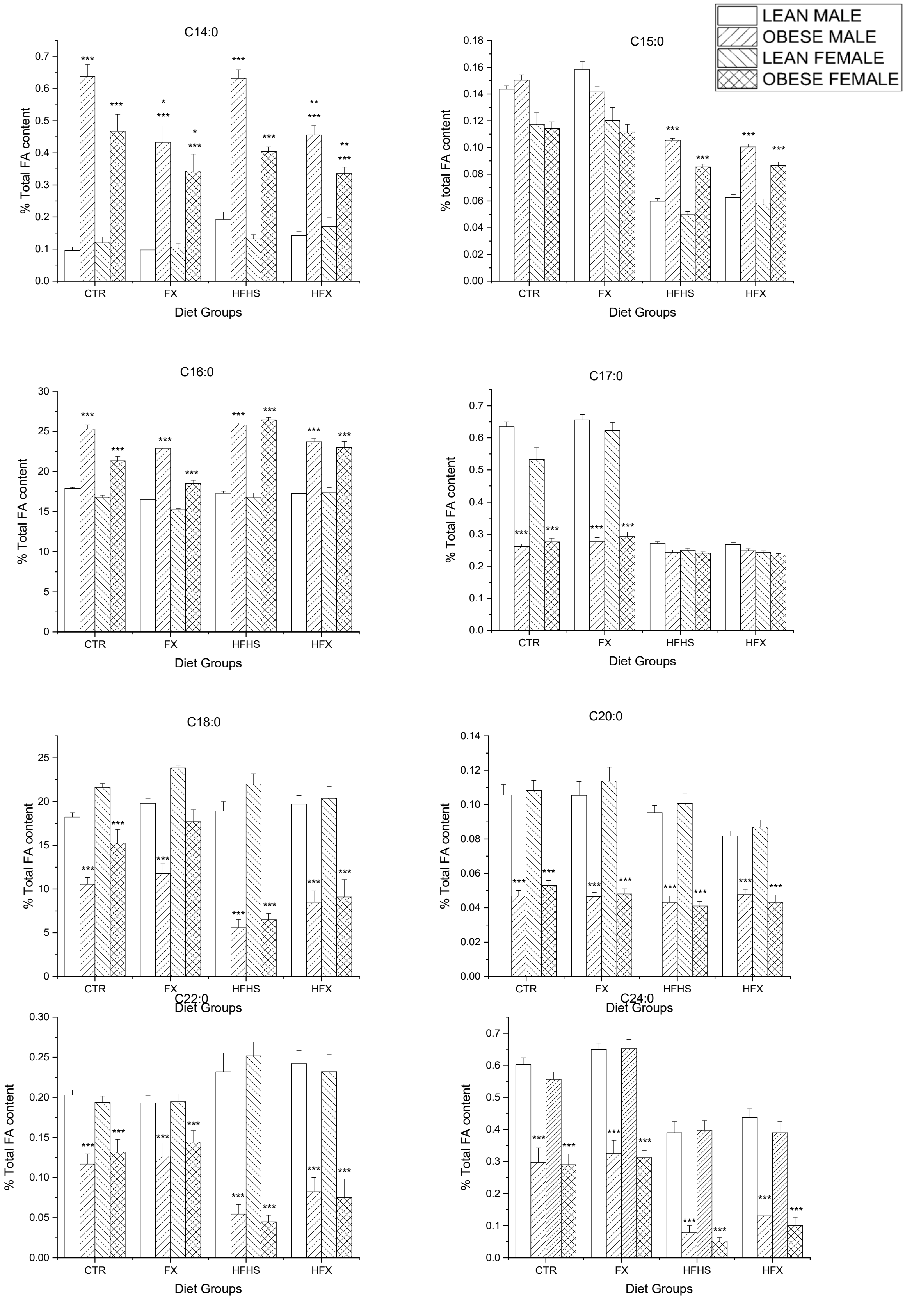
LEAN MALE
OBESE MALE
LEAN FEMALE
OBESE FEMALE
C14:0
C15:0
C16:0
C17:0
C18:0
C20:0
C22:0
C24:0
% Total FA content
% total FA content
CTR
FX
HFHS
HFX
Diet Groups

Figure S1. Composition of liver saturated fatty acids in lean and obese male and female JCR:LA rats as a function of diet. Abbreviations: CTR: control diet; FX: flaxseed supplemented diet; HFHS: high fat and high sucrose supplemented diet; HFX: high fat, high sucrose and flaxseed supplemented diet; *P< 0.05 versus respective control group; **P< 0.05 versus respective HFHS group; ***P<0.05 obese vs lean within same respective dietary group; (n=7 or 8).

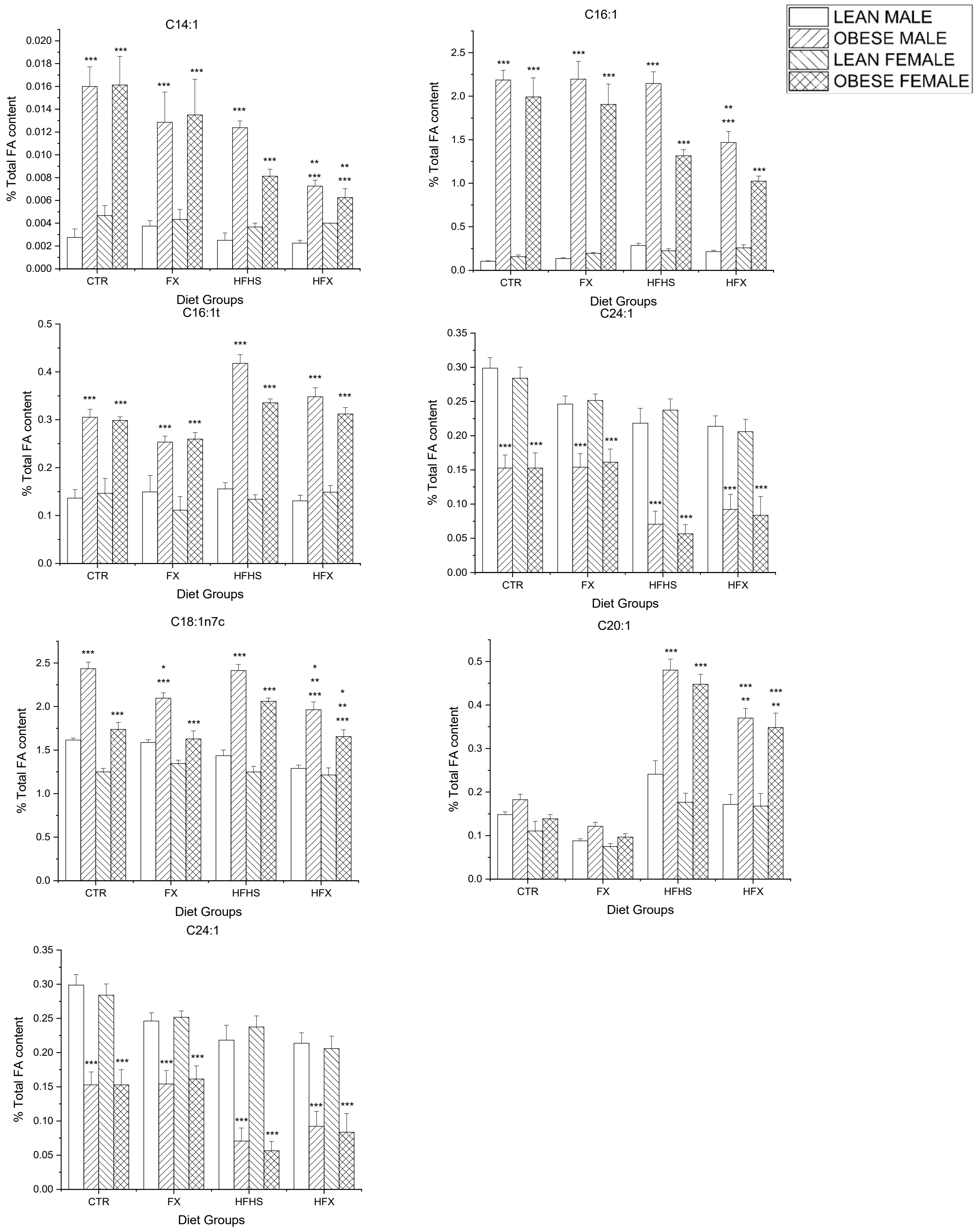

Figure S2. Composition of liver monounsaturated fatty acids in lean and obese male and female JCR:LA rats as a function of diet. Abbreviations: CTR: control diet; FX: flaxseed supplemented diet; HFHS: high fat and high sucrose supplemented diet; HFX: high fat, high sucrose and flaxseed supplemented diet; *$P < 0.05$ versus respective control group; **$P < 0.05$ versus respective HFHS group; ***$P < 0.05$ obese vs lean within same respective dietary group; (n=7 or 8).

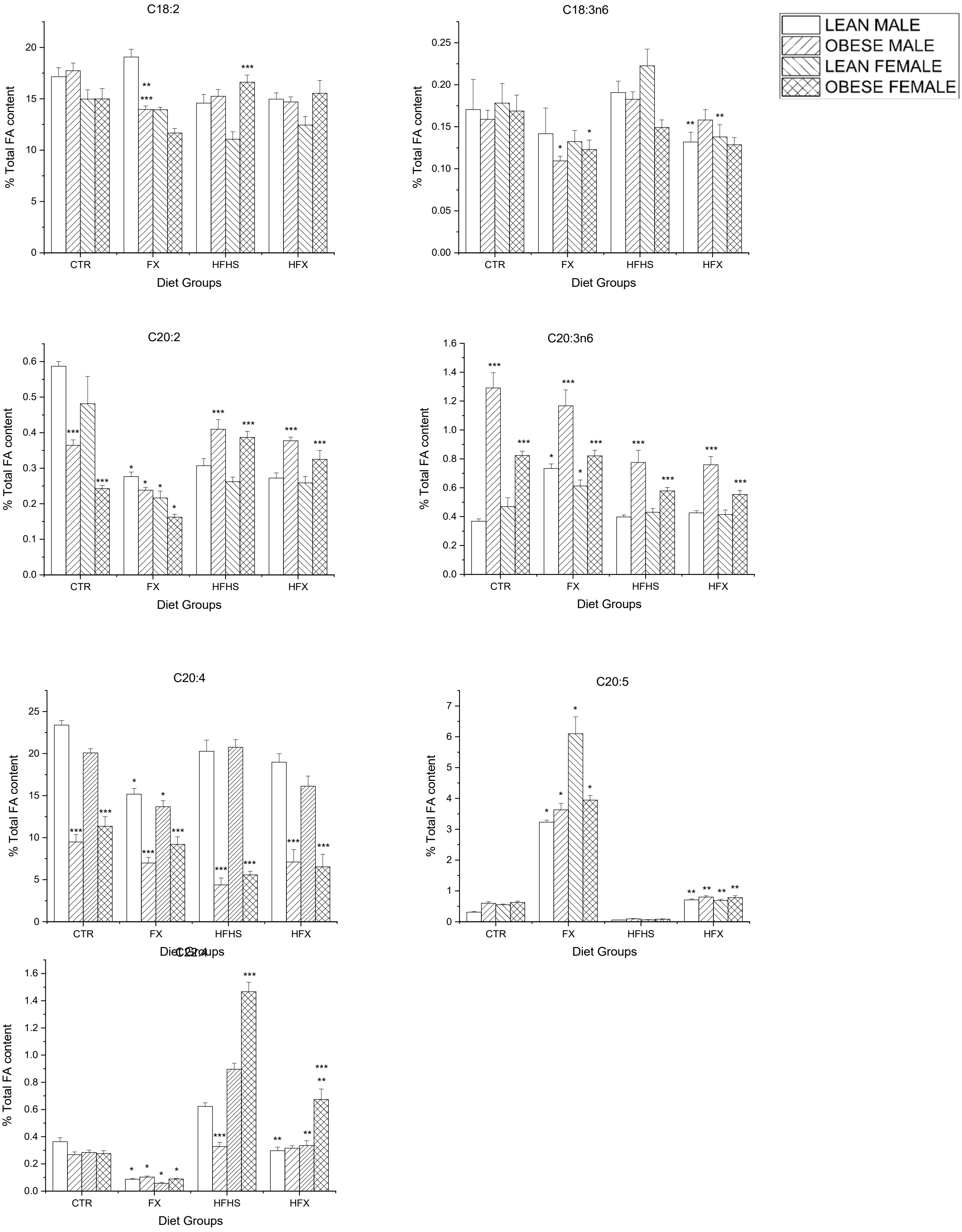

C18:2
C18:3n6
C20:2
C20:3n6
C20:4
C20:5
C22:4
% Total FA content
Diet Groups
CTR
FX
HFHS
HFX
LEAN MALE
OBESE MALE
LEAN FEMALE
OBESE FEMALE

Figure S3. Composition of liver polyunsaturated fatty acids in lean and obese male and female JCR:LA rats as a function of diet. Abbreviations: CTR: control diet; FX: flaxseed supplemented diet; HFHS: high fat and high sucrose supplemented diet; HFX: high fat, high sucrose and flaxseed supplemented diet; *P< 0.05 versus respective control group; **P< 0.05 versus respective HFHS group; ***P<0.05 obese vs lean within same respective dietary group; (n=7 or 8).

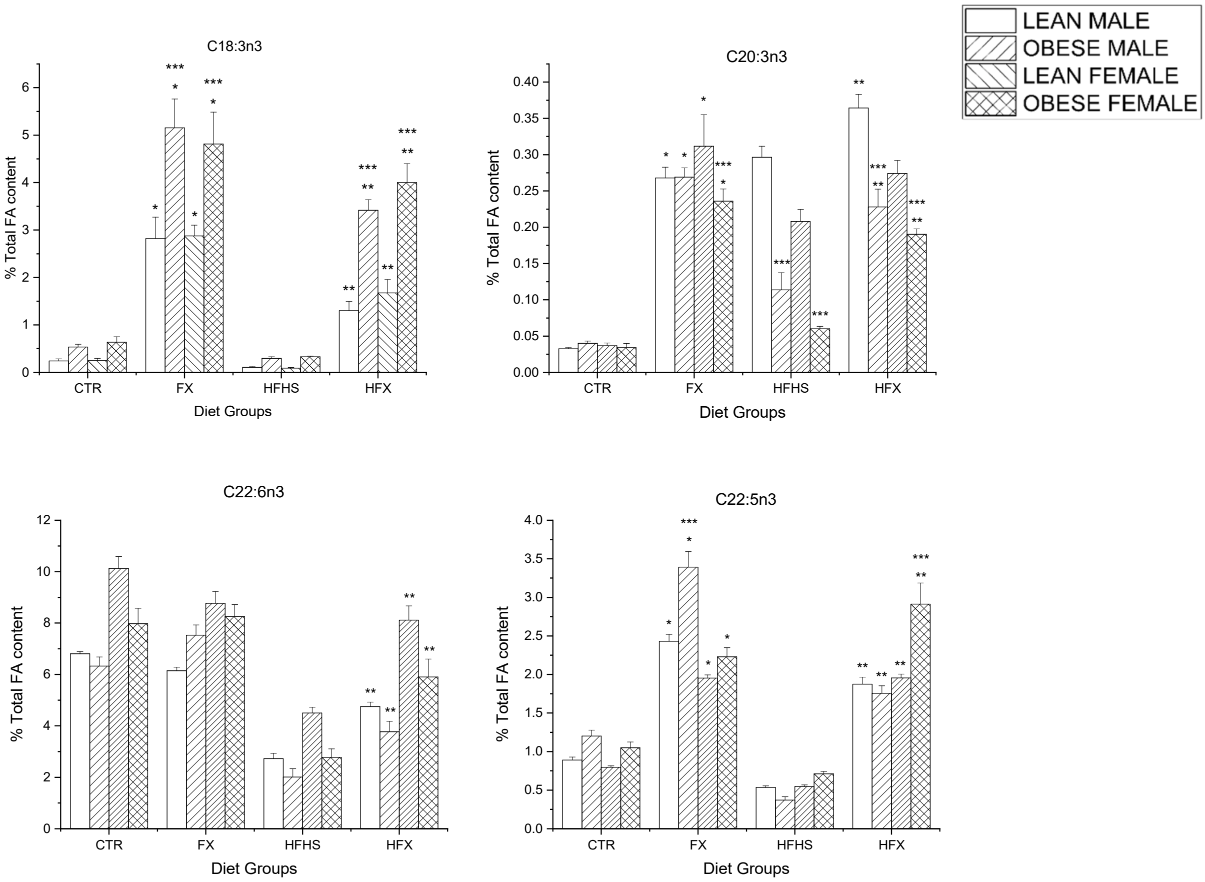

Figure S4. Composition of liver n-3 polyunsaturated fatty acids in lean and obese male and female JCR:LA rats as a function of diet. Abbreviations: CTR: control diet; FX: flaxseed supplemented diet; HFHS: high fat and high sucrose supplemented diet; HFX: high fat, high sucrose and flaxseed supplemented diet; *P< 0.05 versus respective control group; **P< 0.05 versus respective HFHS group; ***P<0.05 obese vs lean within same respective dietary group; (n=7 or 8).

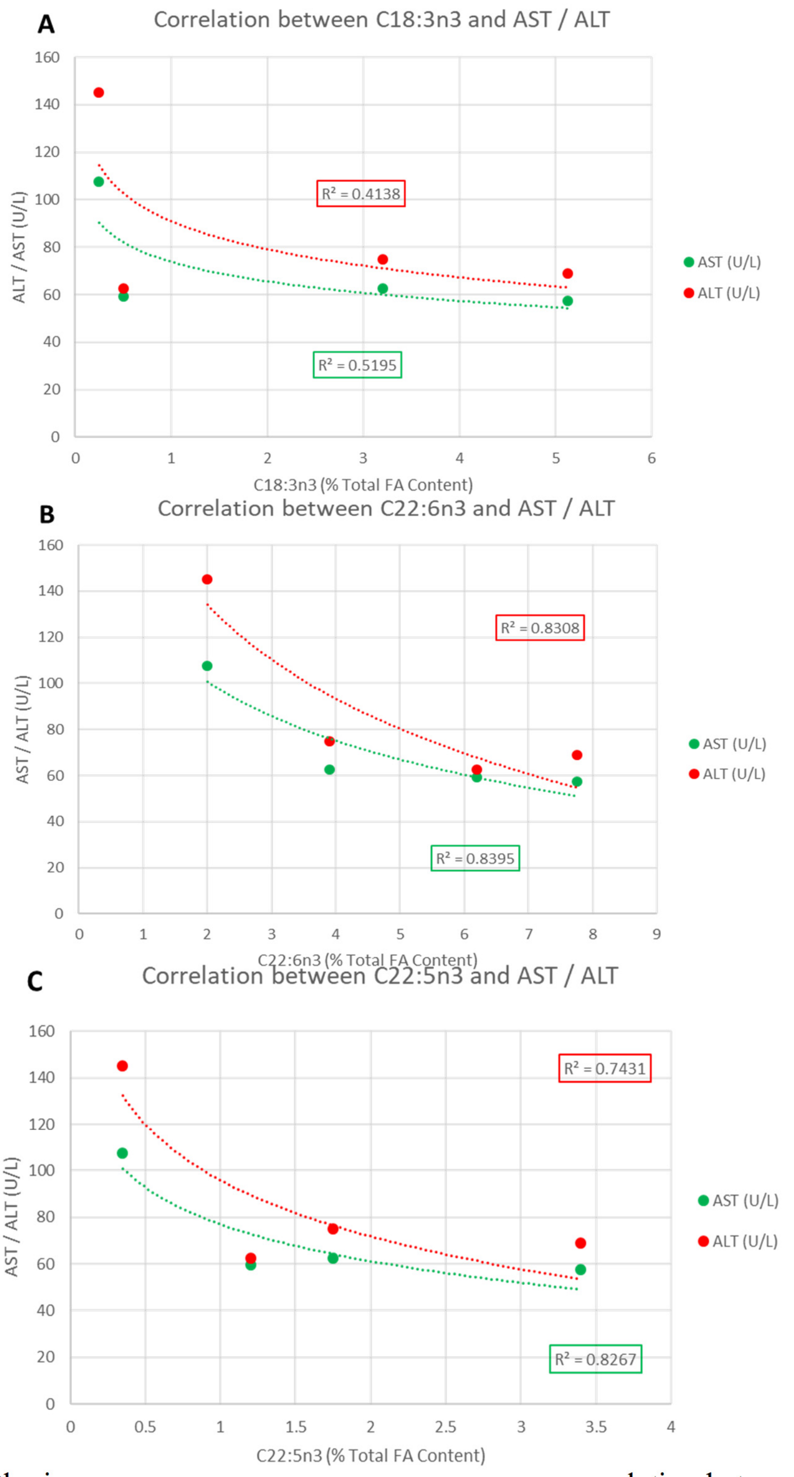

Figure S5. Logarithmic correlation between polyunsaturated fatty acids and ALT and AST release into the plasma. C18:3n3 (A); C22:6n3 (B); C22:5n3 (C).
